# A gene co-expression network predicts functional genes controlling the re-establishment of desiccation tolerance in germinated *Arabidopsis thaliana* seeds

**DOI:** 10.1007/s00425-015-2283-7

**Published:** 2015-03-26

**Authors:** Maria Cecília D. Costa, Karima Righetti, Harm Nijveen, Farzaneh Yazdanpanah, Wilco Ligterink, Julia Buitink, Henk W. M. Hilhorst

**Affiliations:** 1 Wageningen Seed Lab, Laboratory of Plant Physiology, Wageningen University, Droevendaalsesteeg 1, 6708 PB Wageningen, The Netherlands; 2 Institut National de la Recherche Agronomique, UMR 1345 Institut de Recherche en Horticulture et Semences, SFR 4207 Qualité et Santé du Végétal, 49045 Angers, France

**Keywords:** ABA, ABI3, LEA proteins, Transcriptome

## Abstract

**During re-establishment of desiccation tolerance (DT), early events promote initial protection and growth arrest, while late events promote stress adaptation and contribute to survival in the dry state.**

Mature seeds of *Arabidopsis thaliana* are desiccation tolerant, but they lose desiccation tolerance (DT) while progressing to germination. Yet, there is a small developmental window during which DT can be rescued by treatment with abscisic acid (ABA). To gain temporal resolution and identify relevant genes in this process, data from a time series of microarrays were used to build a gene co-expression network. The network has two regions, namely early response (ER) and late response (LR). Genes in the ER region are related to biological processes, such as dormancy, acquisition of DT and drought, amplification of signals, growth arrest and induction of protection mechanisms (such as LEA proteins). Genes in the LR region lead to inhibition of photosynthesis and primary metabolism, promote adaptation to stress conditions and contribute to seed longevity. Phenotyping of 12 hubs in relation to re-establishment of DT with T-DNA insertion lines indicated a significant increase in the ability to re-establish DT compared with the wild-type in the lines *cbsx4*, *at3g53040* and *at4g25580*, suggesting the operation of redundant and compensatory mechanisms. Moreover, we show that re-establishment of DT by polyethylene glycol and ABA occurs through partially overlapping mechanisms. Our data confirm that co-expression network analysis is a valid approach to examine data from time series of transcriptome analysis, as it provides promising insights into biologically relevant relations that help to generate new information about the roles of certain genes for DT.

**Electronic supplementary material:**

The online version of this article (doi:10.1007/s00425-015-2283-7) contains supplementary material, which is available to authorized users.

## Introduction

During seed development, orthodox seeds acquire the remarkable ability to tolerate desiccation. This means that during development, as a part of the maturation phase, these seeds experience slow reduction of their water content to less than 5 % of their dry weight with little or no loss of viability (Ooms et al. 1993). In this dry state, orthodox seeds can survive for years or centuries, which permit their storage and ensure better dispersal (Ramanjulu and Bartels 2002). During seed imbibition and germination, increasing water availability allows metabolic processes to resume, eventually leading to the emergence of the radicle and the associated progressive loss of desiccation tolerance (DT) (Buitink et al. 2003, 2006; Maia et al. 2011). Yet, after radicle protrusion, there is a small developmental window during which DT can be rescued by treatment with an osmoticum (polyethylene glycol, PEG) and/or the plant hormone abscisic acid (ABA) (Buitink et al. 2003; Maia et al. 2011, 2014).

Incubation in PEG induces membrane changes, inhibits radicle growth, down-regulates genes related to energy metabolism and cell wall modification, up-regulates genes related to antioxidant activity, response to stress and seed storage, and induces synthesis of protective molecules, such as non-reducing sugars and certain proteins, such as late embryogenesis abundant (LEA) and heat shock proteins (HSPs) (Buitink et al. 2003, 2006; Maia et al. 2011). Furthermore, genes coding for ABA signal transduction elements and drought/stress-induced transcription factors (TFs) are also up-regulated, leading to the hypothesis of a partial overlap of ABA-dependent and ABA-independent regulatory pathways involved in both drought and DT (Buitink et al. 2003, 2006; Maia et al. 2011). Mutants compromised in ABA sensitivity or synthesis were shown to be still able to produce desiccation-tolerant seeds that, however, are impaired in re-establishment of DT during germination (Maia et al. 2014). Moreover, ABA perception and signaling, more than ABA content, are influencing the ability to re-establish DT (Maia et al. 2014). In previous studies, the re-establishment of DT was brought about by the simultaneous application of cold and osmotic stress (Buitink et al. 2003), or osmotic stress and/or ABA (Maia et al. 2011, 2014). Although treatment of germinated seeds with ABA alone is able to re-establish DT (Maia et al. 2014), the regulatory and mechanistic pathways activated via ABA remain to be elucidated.

Here, we used microarrays to characterize global gene expression during the re-establishment of DT in germinated *A. thaliana* seeds by ABA, and used the data for network analysis of gene co-expression, to gain more resolution on and identify relevant genes in this process. Gene-expression data derived from microarray analysis have been commonly used to provide insight into biological processes at a system-wide level (Wang et al. 2006; Freeman et al. 2007) and is well suited to reconstruct gene regulatory networks to explore potential biological relations (Wang et al. 2006). These networks consist of nodes that represent genes connected by edges that infer co-expression based on a correlation threshold (Freeman et al. 2007). The analysis of this kind of networks is based on the assumption that genes with similar expression patterns have similar functions (Freeman et al. 2007).

In this study, the construction and analysis of a co-expression network proved to be a valid approach to examine data from microarrays of a time series of DT re-establishment by ABA. Using a combination of physiology and transcriptomic approaches, we generated and partially validated a co-expression network, which revealed two major patterns of gene expression during the re-establishment of DT by ABA: an early and a late response. Genes could be divided into those that respond earlier to the incubation in ABA and provide initial protection and signal transduction, and those that respond later and provide adaptation to the stress condition. Validation of the network by functional characterization of genes identified based on the network analysis revealed important roles of some of these genes in the re-establishment of DT in germinated seeds.

## Materials and methods

### Plant growth conditions and germination assays

*Arabidopsis thaliana* plants, accession Columbia (Col-0, N60000), were grown on Rockwool plugs (MM40/40; Grodan), in a climate cell (20 °C day, 18 °C night), under 16 h of light, and watered with Hyponex nutrient solution (1 g l^−1^, http://www.hyponex.co.jp). Seeds were bulk harvested in three replicates of at least two plants. Seeds used in germination assays were cold stratified for 72 h at 4 °C in 9-cm Petri dishes on two layers of blue filter paper (Blue Blotter Paper, Anchor Paper Company, http://www.seedpaper.com) and 10 ml of distilled water. After stratification, seeds were transferred to germination cabinets with constant white light at 22 °C.

### Re-establishment of DT using ABA

To assess the re-establishment of DT using ABA, germinated seeds at the stage of radicle protrusion (stage II, Maia et al. 2011) were incubated for a maximum of 3 days in 6-cm Petri dishes containing 1.3 ml of an ABA solution (10 µM) on two sheets of white filter paper (grade 3hw, Biolab Products, Sartorius Stedim Biotec) in the dark at 20 °C. The incubation in ABA was done in the dark to reduce oxidative damage. After incubation, seeds were rinsed in distilled water, transferred to new Petri dishes with one dry sheet of white filter paper and dried for 3 days at 40 % relative humidity (RH) at 20 °C, resulting in water content levels as low as 0.08 g H_2_O g^−1^ dry weight. After drying, seeds were pre-humidified in air of 100 % RH for 24 h at 22 °C in the dark and subsequently rehydrated in water on a Copenhagen Table under a 12/12 h dark/light regime at 20 °C. Germinated seeds were evaluated according to the survival of their primary root, presence of green and fully expanded cotyledons (cotyledon survival) and growth resumption with both green and fully expanded cotyledons and development of a root system (seedling survival).

### Longevity of germinated seeds

Longevity of germinated seeds was evaluated by an accelerated aging test after incubation in ABA for 24 h and 72 h, drying for 3 days at 40 % RH, storage for 24 h at 80 % RH and 40 °C in the dark and rehydration. The parameters evaluated were survival of the primary root, cotyledons survival and seedling survival.

### RNA extraction and microarray hybridization

Seeds at the stage of radicle protrusion (control) and these seeds after four periods (2, 12, 24 and 72 h) of incubation in ABA were used for RNA extraction. Total RNA was extracted from three replicates of approximately 1000 germinated seeds for each time point following a modified hot borate protocol (Wan and Wilkins 1994; Maia et al. 2011). The seeds were ground and mixed with 800 µl of extraction buffer (0.2 N Na borate decahydrate (Borax), 30 mM EGTA, 1 % SDS, 1 % Na deoxycolate) containing 1.76 mg DTT and 52.8 mg PVP40, and heated to 80 °C. In the next step, 4 mg proteinase K was added to this solution before incubation for 15 min at 42 °C. After the addition of 64 µl of 2 M KCL, the samples were incubated on ice for 30 min and subsequently centrifuged for 20 min at 12,000*g*. The supernatant was transferred to a new tube, 260 µl of ice-cold 8 M LiCl was added, and the tubes were incubated overnight on ice. After centrifugation at 4 °C for 20 min at 12,000*g*, the pellets were washed with 750 ml of ice-cold 2 M LiCl and re-suspended in 100 µl milliQ water. The samples were phenol–chloroform extracted, DNAse treated (RQ1 DNase, Promega) and further purified with RNeasy spin columns (Qiagen) according to the manufacturer’s instructions. RNA quality and concentration were assessed by agarose gel electrophoresis and a NanoDrop ND-1000 spectophotometer (Nanodrop^®^ Technologies, Wilmington, DE, USA). RNA was processed for the use on Affymetrix ARAGene 1.1ST Arrays as described by the manufacturer. In brief, reverse transcription was employed to generate double-stranded cDNA that was in vitro transcribed to biotinylated cRNA. The biotinylated cRNA was used for hybridization. The Affymetrix HWS Kit for GeneTitan (part nr. 901622) was used for hybridization, washing and staining of the array plates. The array plates were scanned using the Affymetrix Command Console v3.2 software.

All data are MIAME compliant as detailed on the MGED Society website http://www.mged.org/Workgroups/MIAME/miame.html. The microarray data have been deposited on the NCBI’s Gene Expression Omnibus (Edgar et al. 2002) and is accessible through the GEO Series accession number GSE62876.

### Microarray analysis

Signal intensities were extracted and analyzed using the Bioconductor packages of R (Gentleman et al. 2004). The data were normalized using the RMA algorithm (Irizarry et al. 2003) with the TAIRG v17 cdf file (http://www.brainarray.mbni.med.umich.edu).

To equalize background noise, gene expression values less than four were replaced with four (Dekkers et al. 2013). After this transformation, fold changes were calculated comparing each time-point with the control (germinated seeds at the stage of radicle protrusion non-treated with ABA). A gene was considered differentially expressed (the differentially expressed genes: DEGs) if the difference between its mean expression in at least one time-point and the control was statistically significant at *P* ≤ 0.01 after application of linear modeling with thresholds for absolute fold change of 2.0 (on a log_2_ scale).

The resulting gene set was used for an over-representation analysis (ORA) to recover over-represented biological processes (using Bonferroni-corrected *P* value at 0.05) based on gene ontologies using Gene Trail (Keller et al. 2008). The term’s semantic distance with respect to other semantically close terms (“Dispensability”) was calculated using the online tool ReviGO (Supek et al. 2011) and redundant gene ontology (GO) terms were removed applying a cut-off of <0.1 for this “Dispensability” value.

### Network construction and analysis

Pearson correlation coefficients between all pairs of DEGs were calculated. A table with correlation coefficient values was exported to Cytoscape v.2.8.2 (Smoot et al. 2011) and correlation coefficients above a threshold of 0.97 (determined according to Freeman et al. 2007) were used to filter the connections that were used to determine the edges between nodes in the network. The resulting network was displayed with a yGraph Organic layout. The Cytoscape plug-in NetworkAnalyzer (Assenov et al. 2008) was used to compute node degree. The 100 nodes with the highest degrees were considered “hubs”.

Self-organizing maps (SOMs) were calculated using GeneMaths software (version 2.1, Applied Maths BVBA, Sint-Martens-Latem, Belgium) by importing gene expression data and mapping them into six groups (2 × 3 node format) that provided optimal representation of gene expression patterns in a reasonably small number of independent bins.

### Mutant analysis

T-DNA insertion lines (Suppl. Table S1; Supp. Fig. S1) for selected genes were obtained from the Nottingham Arabidopsis Stock Centre (Scholl et al. 2000). Plants with homozygous T-DNA insertions were selected and confirmed using PCR and grown as described above for wild-type plants. Seeds were bulk-harvested in three replicates of at least two plants. Germinated seeds at the stage of appearance of first root hairs (stage IV, Maia et al. 2011) were phenotyped to evaluate the ability to re-establish DT after treatment with PEG as described by Maia et al. (2011). The evaluated parameters were survival of their primary root, cotyledon survival and seedling survival.

Seeds were also phenotyped for seed dormancy, longevity, and vigor. Seed dormancy was evaluated as the number of days of Seed Dry Storage required to reach 50 % germination (DSDS50) (Alonso-Blanco et al. 2003). Seed longevity was estimated based on an accelerated aging assay (germination percentage after storage for 6 days at 80 % RH and 40 °C in the dark) (Bentsink et al. 2000). Seed vigor was measured as the ability of seeds to germinate at high temperature (at 33 °C) or on NaCl (130 mM). Germination experiments and scoring of germination were performed with the GERMINATOR as described by Joosen et al. (2010) in a fully randomized setup. For each measurement, three replicates of 40–60 seeds per seed batch were used.

## Results

### Re-establishment of DT in germinated *A. thaliana* seeds

Mature *A. thaliana* seeds are desiccation tolerant and imbibition and progression into germination change their status to desiccation sensitive already at the phase of testa rupture (Maia et al. 2011). However, a treatment with ABA fully rescues DT in germinated seeds till the phase of radicle protrusion (Maia et al. 2014). In subsequent developmental stages, DT can no longer be rescued in all organs (Maia et al. 2014).

**Fig. 1:**
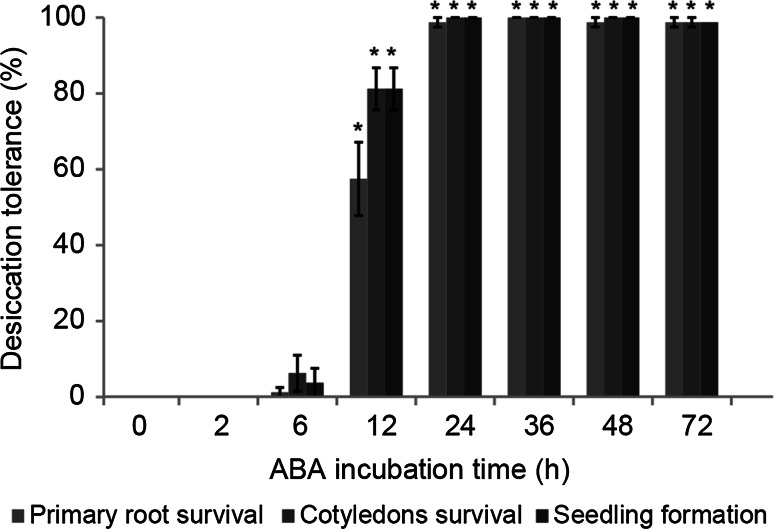
Re-induction of desiccation tolerance in germinated *A. thaliana* seeds at the stage of radicle protrusion during incubation in ABA. *Vertical bars* represent standard error. *Asterisks* indicate significant differences at *P* ≤ 0.01 comparing each parameter in each time-point and the control

**Fig. 2:**
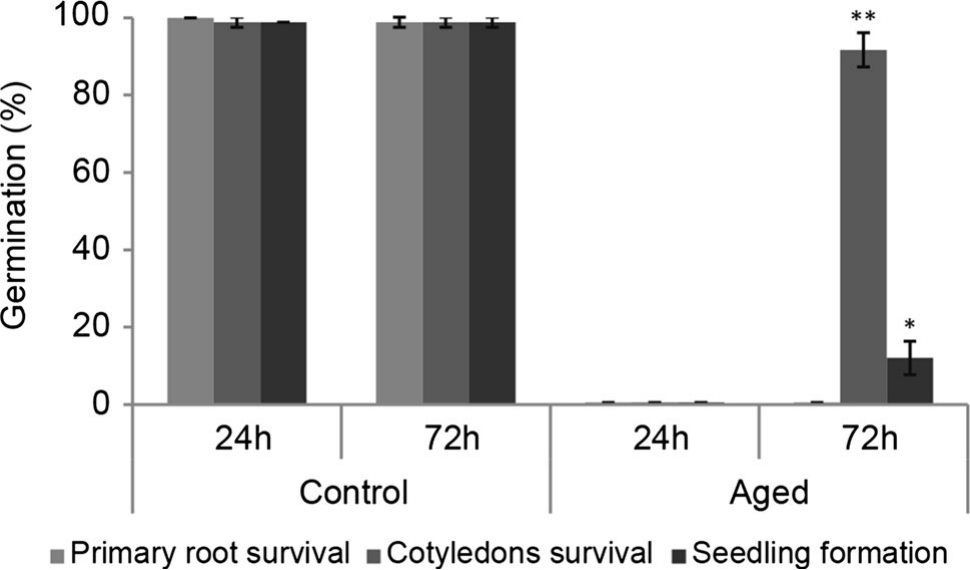
Longevity of germinated *A. thaliana* seeds at the stage of radicle protrusion incubated in ABA for 24 or 72 h. Longevity was estimated as germination percentage after aging (24 h of storage at 80 % RH and 40 °C). Control germinated seeds were incubated in ABA, but not aged. *Asterisks* indicate significant differences at *P* ≤ 0.05 for one, *P* ≤ 0.01 for *two asterisks* comparing 24 h and 72 of incubation in ABA for each parameter in control and aged seeds separated

After 12 h, the treatment with ABA had already led to the re-establishment of DT in 80 % of germinated *A. thaliana* seeds at the stage of radicle protrusion (Fig. 1). After 24 h, all the seeds had re-established DT. To further assess the capacity of survival in the dry state, storability was determined by an accelerated aging test. Survival rates after the accelerated aging test were lower for seeds incubated in ABA for 24 h than for seeds incubated in ABA for 72 h (Fig. 2).

To gain temporal resolution of the changes in gene expression induced by incubation in ABA, gene expression analysis was performed on a time series. Based on the results shown in Fig. 1, five time-points were chosen for microarray analysis: 0 (control), 2, 12, 24 and 72 h of incubation in ABA.

**Fig. 3:**
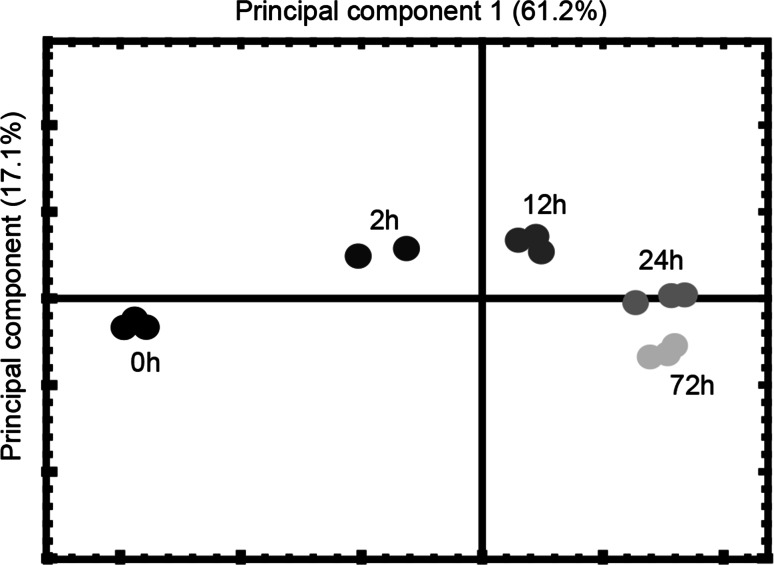
Principal component analysis of microarray data on different time points of ABA incubation. The *different time* *points* are indicated by *different shades* of *gray*

Principal component analysis was used to compare global changes between the transcriptomes of the different time-points and to evaluate group clustering. One replicate of time-point 2 h was a statistically significant outlier (data not shown) and was removed from further analysis. Without this replicate, the first principal component described 61.2 % of the variation (Fig. 3).

In total, 1177 genes were considered differentially expressed in response to the ABA treatment for at least one time-point as compared to the control. The number of DEGs was the lowest after 2 h of incubation and increased until 24 h, after which no substantial changes occurred until 72 h of incubation (Table 1). To verify the accuracy of the microarray data, the expression of 20 genes with different expression patterns were also analyzed by qPCR. Both microarray data and qPCR analysis showed comparable trends (Suppl. Fig. S2).

**Table 1:** Distribution of DEGs after statistical analysis comparing each time-point against the control (not treated with ABA)

Time-point (h)	Accumulating	Declining	Total
2	189	111	300
12	297	290	587
24	444	439	883
72	473	394	867

Thresholds were 2.0 for absolute fold change (on a log_2_ scale) and 0.01 for *P* value

### Over-representation analysis (ORA) of functional GO categories

**Fig. 4:**
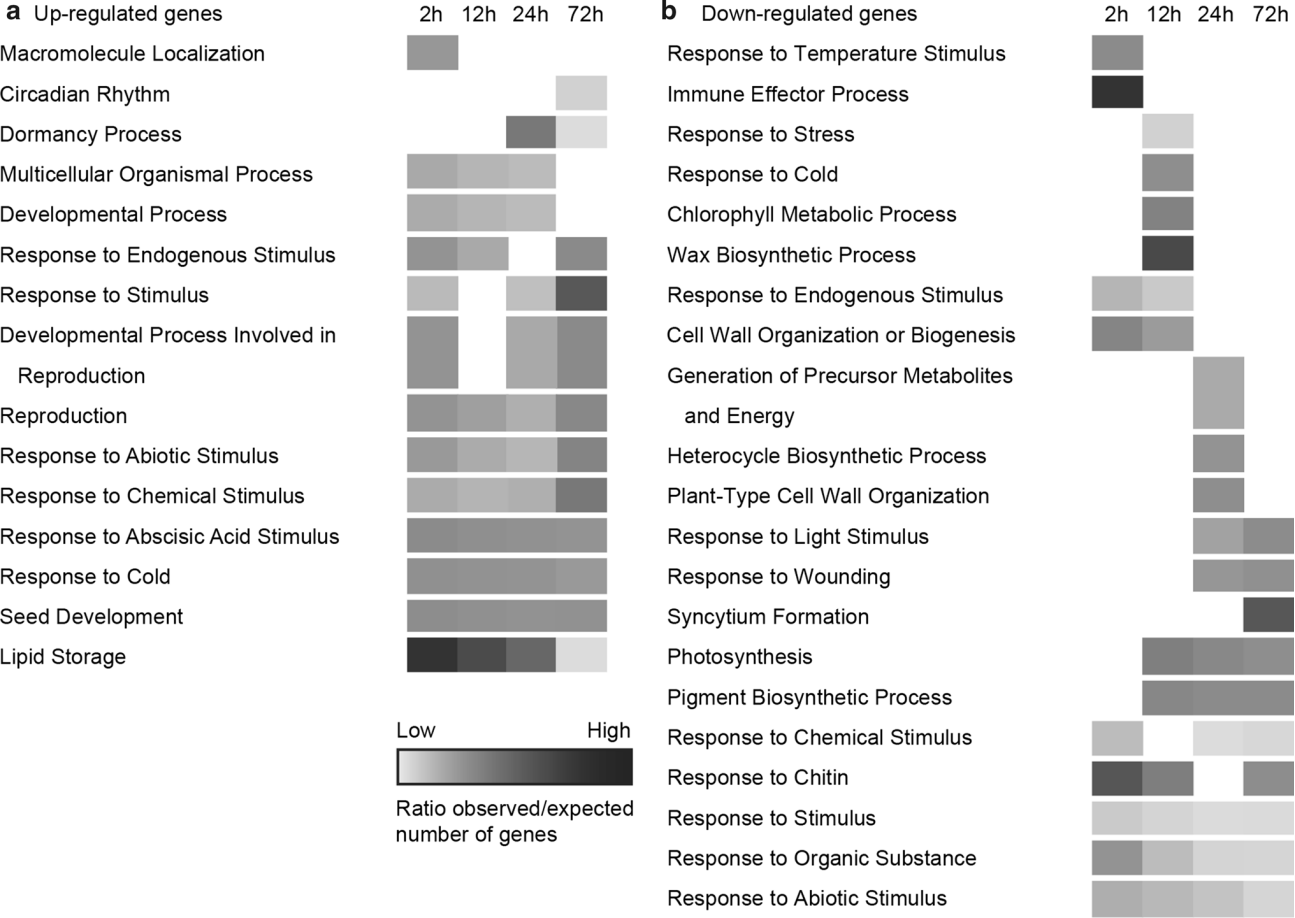
Heat-map of ORA of biological processes on differences between different time-points during incubation of germinated *A. thaliana* seeds in ABA relative to the control (non-treated with ABA). Functional classes were determined using Gene Trail and ReviGO. *P* values were adjusted for multiple testing by the Bonferroni method applying a cut-off of <0.05. **a** Results for genes with accumulating transcript levels in each time-point compared to the control. **b** Results for genes with declining transcript levels in each time-point compared to the control. *Shades* of *gray* indicate the extent (ratio of expected to observed number of genes) to which the category was overrepresented in each comparison

ORA was used to get an overview of the enriched functional GO categories comparing the expression data of genes from each time-point relative to the control. Among the genes with accumulating transcript levels, the category lipid storage was highly overrepresented after 2 h of incubation in ABA but, over time, this category became less overrepresented (Fig. 4). The category dormancy process is over-represented in the genes with accumulating transcript levels only after 24 h of incubation, when DT was fully re-established. Within the genes represented by declining transcript levels, categories related to cell wall (wax biosynthetic process, cell wall organization or biogenesis, plant-type cell wall organization, and syncytium formation) were over-represented. Genes in these categories are mainly involved in depolymerisation of cell wall components during the beginning of storage mobilization (Sreenivasulu et al. 2008). Categories related to photosynthesis and metabolism (chlorophyll metabolic process, generation of precursor metabolites and energy, and photosynthesis) were also over-represented within the DEGs represented by declining transcript levels after 12 and 24 h, when 80 and 100 % of the seeds had re-established DT, respectively. Overall, this analysis revealed significant accumulation of transcripts of genes involved in protection, response to stimulus, seed development, and seed dormancy and decline of transcripts of genes related to cell growth, photosynthesis and response to stimulus (Fig. 4).

### Gene co-expression network

**Fig. 5:**
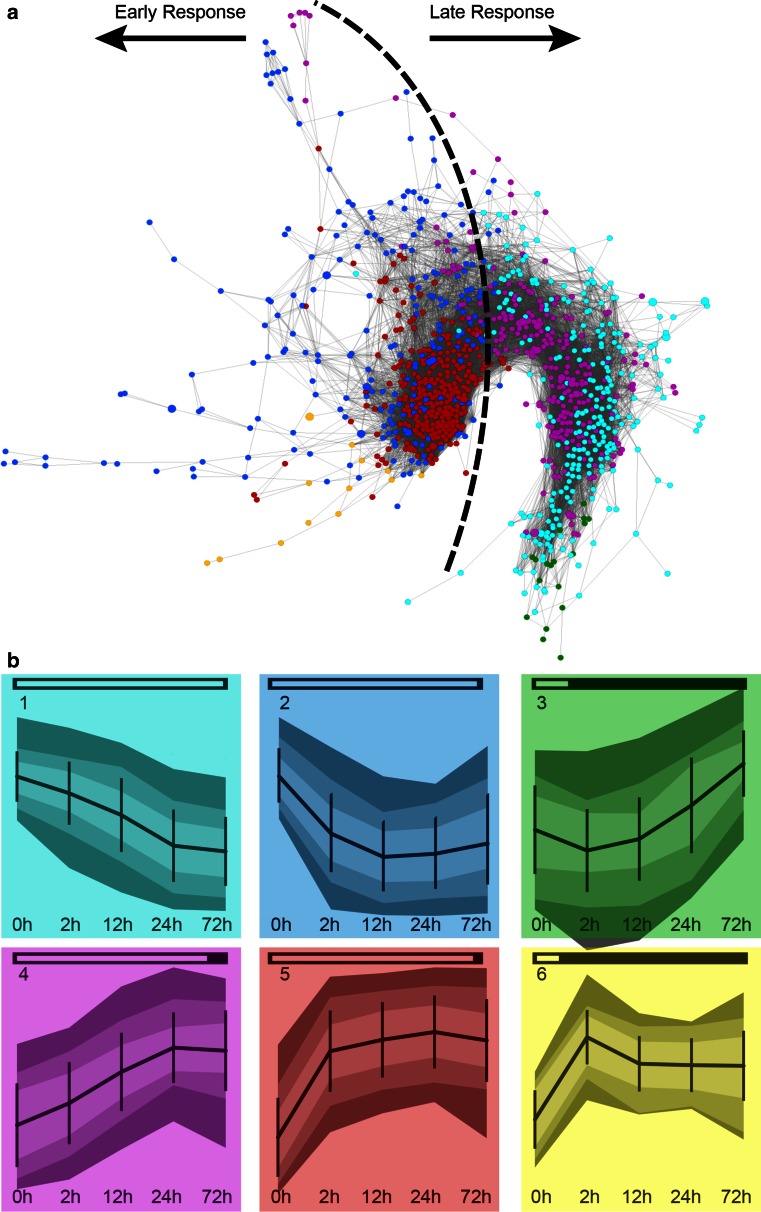
Gene co-expression network (**a**) and SOM (**b**) of transcriptomes of germinated *A. thaliana* seeds incubated in ABA for different intervals. The network is visualized using an yGraph Organic layout in Cytoscape and the temporal analysis of modules was obtained by *coloring* each node by the profile specific for the SOM groups. The *dashed line* and the *arrows* in the network indicate the two regions, namely early response and late response. For the SOM, the probe sets were grouped into six clustered patterns. *Horizontal bars* at the top of each graph represent the number of probe sets belonging to a certain SOM bin. The SOM bins having the highest number of genes have the largest bar. *Vertical bars* represent standard deviation in average expression of the genes in each time point per group. The *three color* *shades* in each graph ranging from dark to light correspond to the 98th, 90th and 75th percentiles, respectively

Transcriptional studies generate vast amounts of gene expression data. This type of data can be used to build co-expression networks which help to study coordinated gene expression and to identify key genes, functional modules or relations between the network structure, and additional information (Mutwil et al. 2010; Villa-Vialaneix et al. 2013). Using Cytoscape, we constructed a gene co-expression network from the correlation coefficients calculated between all pairs of DEGs. The DEGs are represented as nodes connected by edges that model significant correlation coefficients (Villa-Vialaneix et al. 2013). The network was built from 1083 genes/nodes (Suppl. Table S2) completely connected by 35,296 edges, meaning that any node could be reached from any other node by a path passing along the edges (Villa-Vialaneix et al. 2013) and visualized using an organic layout in Cytoscape (Fig. 5a).

SOMs can be used to categorize gene expression data into groups that show similar gene expression profiles and thereby may also contain functionally related genes (Törönen et al. 1999). We colored each node of the co-expression network by the profile group according to the SOM (Fig. 5b). In this way, two main regions of highly interconnected transcripts were identified: early response (ER) and late response (LR). The ER region is the most tightly co-regulated one with nodes representing genes with a sharp increase or decrease in expression after 2 h of incubation in ABA. This region concentrates the hubs of the network, meaning that it contains all 100 nodes with the highest degrees (number of nodes directly connected to a given node, ranging from 165 to 209). The LR region contains genes with slow increase or decrease in expression during incubation in ABA.

To assess the cross-link between re-establishment of DT with ABA and seed and stress-related processes, we projected sets of DEGs on the network comparing: dormancy (dormant seeds vs. after-ripened seeds; Cadman et al. 2006, Fig. 6a); DT acquisition (the cotyledon stage vs. the post mature-green stage of seed development; Le et al. 2010, Fig. 6b); re-establishment of DT with PEG [germinated seeds at the stage of radicle protrusion before (desiccation sensitive) vs. after (desiccation tolerant) treatment with PEG; Maia et al. 2011, Fig. 6c]; and drought (control plants vs. plants subjected to drought for 10 h; Matsui et al. 2008, Fig. 6d). Genes with increased transcript levels in these comparisons locate mainly in the ER region of the network, while genes with decreased transcript levels locate mainly in the LR region. Moreover, we found an overlap of 16 genes differentially expressed in relation to all these physiological processes (Table 2).

**Fig. 6:**
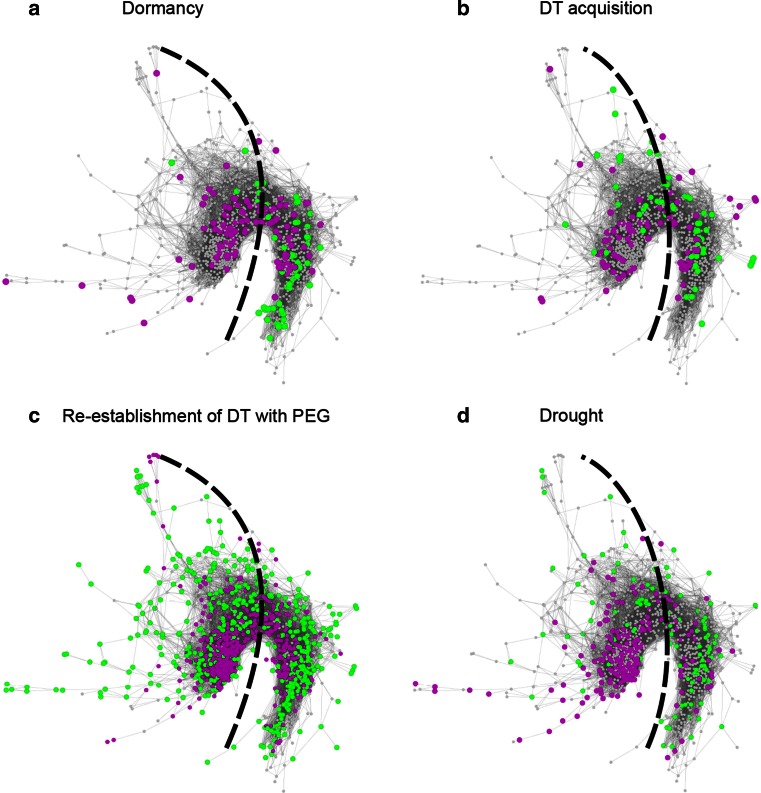
Gene co-expression network of incubation in ABA of germinated *A. thaliana* seeds. Nodes are *colored* according to genes with accumulating (*purple*) or declining (*green*) transcript levels in different datasets comparing: **a** Dormancy (dormant seeds vs. after-ripened seeds; Cadman et al. 2006). **b** DT acquisition (cotyledon stage vs. post mature-green stage of seed development; Le et al. 2010). **c** Re-establishment of DT with PEG (germinated seeds in the stage of radicle protrusion before (desiccation sensitive) vs. after (desiccation tolerant) treatment with PEG; Maia et al. 2011). **d** Drought (control plants vs. plants dried for 10 h; Matsui et al. 2008). The *dashed line* separates the early response (*left*) and the late response (*right*) region

**Table 2:** Genes differentially expressed in all the following processes: dormancy (dormant seeds vs. after-ripened seeds; Cadman et al. 2006); DT acquisition (cotyledon stage vs. post mature-green stage of seed development; Le et al. 2010); re-establishment of DT with PEG (germinated seeds in the stage of radicle protrusion before (desiccation sensitive) vs. after (desiccation tolerant) treatment with PEG; Maia et al. 2011); and drought (control plants vs. plants dried for 10 h; Matsui et al. 2008)

AGI	Annotation	AGI	Annotation
AT1G05340	Unknown protein	AT2G16430	PURPLE ACID PHOSPHATASE 10 (PAP10)
AT1G13640	Phosphatidylinositol 3- and 4-kinase family protein	AT2G31350	GLYOXALASE 2-5 (GLX2-5)
AT1G14530	TOM THREE HOMOLOG 1 (THH1)	AT2G33830	Dormancy/auxin-associated family protein
AT1G15330	Cystathionine beta-synthase protein	AT3G03310	LECITHIN:CHOLESTEROL ACYLTRANSFERASE 3 (LCAT3)
AT1G15740	Leucine-rich repeat family protein	AT3G20250	PUMILIO 5 (PUM5)
AT1G21680	DPP6 N-terminal domain-like protein	AT3G26580	Tetratricopeptide repeat (TPR)-like superfamily protein
AT1G55530	RING/U-box superfamily protein	AT4G05390	ROOT FNR 1 (RFNR1)
AT1G64660	METHIONINE GAMMA-LYASE (MGL)	AT4G29190	OXIDATION-RELATED ZINC FINGER 2 (OZF2)

After the projection on the network of the DEGs in the re-establishment of DT with PEG, 84 % of the nodes were highlighted, with a clear overlap between genes with increasing and decreasing transcript levels in both processes (re-establishment of DT by ABA and PEG) (Fig. 6c). A comparison between over-represented GO categories in DEGs with accumulating transcript levels in response to incubation in ABA and in PEG revealed a large overlap in categories related to response to stimulus and seed development (Maia et al. 2011). However, the categories circadian rhythm, dormancy process and macromolecule localization were over-represented only in response to incubation in ABA, while categories related to respiration were over-represented only in response to incubation in PEG. ABA regulates the expression of genes related to circadian clock through TFs such as ABI5, AP2/ERF and NAC (Fujita et al. 2011), and the induction and maintenance of dormancy (Finch-Savage and Leubner-Metzger 2006). The osmotic stress and a possible hypoxia caused by the incubation in PEG may have induced the expression of genes related to respiration, such as *AT1G19530*, *AT1G33055*, *AT3G10020*, *ADH1*, leading to the over-representation of the categories anaerobic respiration and cellular respiration (Maia et al. 2011). Considering DEGs with declining transcripts, GO categories related to cell wall, photosynthesis and response to stimulus were over-represented in response to both incubation in ABA and in PEG (Maia et al. 2011). The categories heterocycle biosynthetic process and syncytium were over-represented only in response to incubation in ABA, while the categories fatty acid metabolic process and sulfur metabolic process were over-represented only in response to incubation in PEG. The over-representation of these categories is an indication that incubation in either ABA or PEG leads to growth arrest by affecting different genes. Incubation in ABA reduces abundance of transcripts related to expansins (*ATEXPA1, ATEXPA8* and *ATEXPA 10*), extensins (AT2G43150, AT3G28550 and AT3G54580) and certain photosynthesis-related genes (*APT3, APT5, GUN4, GUN5, HEMA1* and *PORB*) that are not significantly affected by PEG treatment, while PEG reduced the abundance of gene transcripts related to fatty-acid metabolism (*AT5G10160, ACP4, HCD1, OPR1* and *OPR2*) and related to the synthesis of compounds that contain sulfur (*ATGSTU27*, *CYP79B2*, *CYP83B1*, *MAT3* and *MTO3*), such as the amino acid cysteine.

**Fig. 7:**
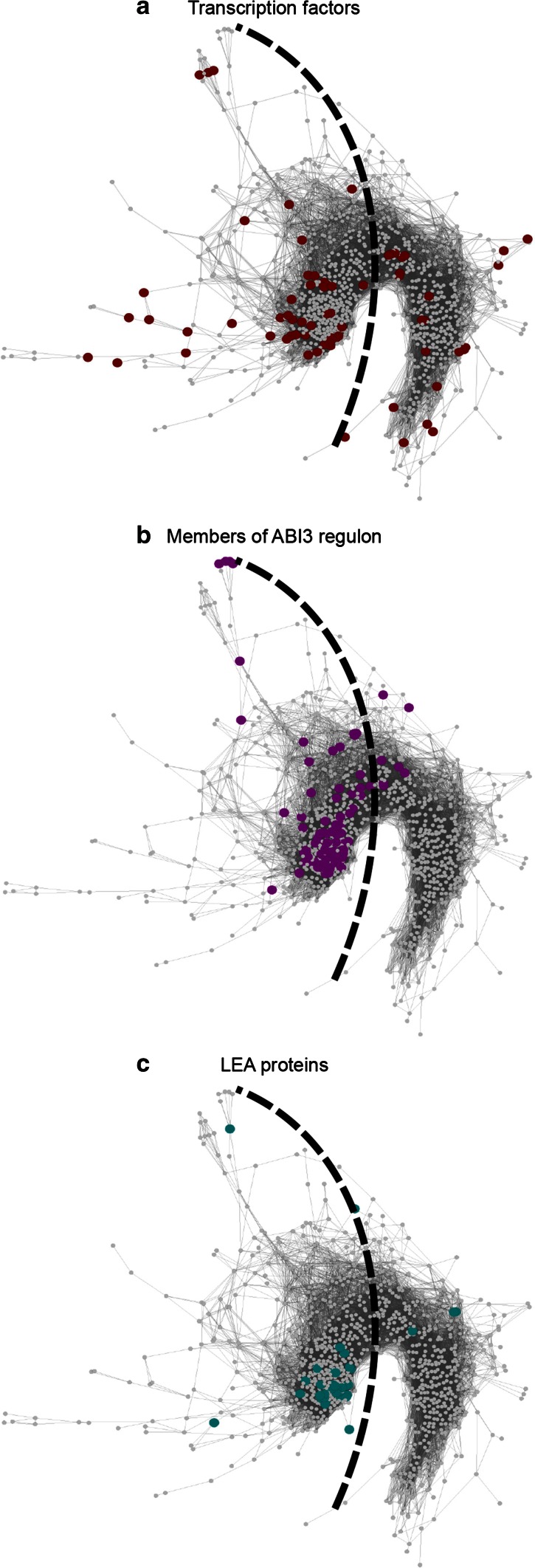
Gene co-expression network of germinated *A. thaliana* seeds incubated in ABA. Nodes are *colored* according to genes encoding transcription factors (**a**), members of the ABI3 regulon (**b**), and LEA proteins (**c**). The *dashed line* separates the early response (*left*) and the late response (*right*) region of the network

Considering that in plant genomes many TFs are master regulators of signaling and regulatory pathways of stress adaptation and act in an ABA-dependent manner (Lindemose et al. 2013), we searched for the presence of known and predicted TFs as listed in the database of *Arabidopsis* transcription factors (DATF, http://www.datf.cbi.pku.edu.cn, Guo et al. 2005) in our network. From the 2290 TFs gene model identifiers available in the DATF, we found 69 in our network (Fig. 7a). Of these, 46 are located in the ER region and are mainly related to response and tolerance to abiotic stress, such as *Abscisic acid insensitive* 5 (*ABI5*), members of the APETALA 2/ethylene-responsive element-binding factor (AP2/ERF) family (*AT5G18450*, *AT5G51190* and *ERF5*), members of the NAM/ATF1/CUC2 (NAC) class (*NAC032*, *NAC048*, *NAC053*, *NAC060*, *NAC083* and *NAC089*) and WRKY TFs (*WRKY 18*, *WRKY29* and *WRKY36*). TFs located in the LR region are mainly related to abiotic stress tolerance, growth regulation and light signaling pathways, such as *ABRE*-*binding factor 1* (*ABF1*), *enhanced* *Em levels* (*EEL*), *LATERAL ORGAN BOUNDARY DOMAIN 41* (*LBD41*), *LATE ELONGATED HYPOCOTYL* (*LHY*), *PHYTOCHROME*-*INTERACTING FACTOR*-*LIKE 2* (*PIL2*), *PHYTOCHROME INTERACTING FACTOR 4* (*PIF4*), *PSEUDO*-*RESPONSE REGULATORS* (*PRR5*), *TIMING OF CAB EXPRESSION 1* (*TOC1*) and *WUSCHEL RELATED HOMEOBOX 12* (*WOX12*).

The TF *ABI3* is a key regulator of seed development and maturation that, together with ABA, plays an essential role in the protection of embryonic structures from desiccation (Mönke et al. 2012). Targets of ABI3 include genes with a protective role in DT, such as LEA genes. We projected the set of target genes from the ABI3 regulon (Mönke et al. 2012) (Fig. 7b) and the set of genes from the inventory of all LEA proteins identified in the *A. thaliana* genome on our network (Bies-Ethève et al. 2008) (Fig. 7c). Most of the genes representing the ABI3 regulon and LEA proteins are concentrated in the ER region of the network. Within the hubs identified in the network, genes of the ABI3 regulon and LEA genes are significantly (*P* value <0.01) enriched with 44 and 19 genes respectively.

We selected 12 hub genes that are members of the ABI3 regulon, six of which are LEA genes, and investigated their function in the re-establishment of DT and seed-related traits such as dormancy, longevity and vigor (Suppl. Table S3). These genes were chosen based on the indications of their involvement in DT from ongoing studies in our laboratory. The evaluation of seed-related traits is important since the acquisition of DT by seeds requires a series of mechanisms that are also necessary for seed survival in the dry state (Kalemba and Pukacka 2011; Delahaie et al. 2013) and the disruption of one or more of these mechanisms can impact other aspects important for the completion of germination. We characterized T-DNA insertion lines for each of the aforementioned 12 genes. *ECP63*, *AT2G42560*, *AT3G15670*, *AT3G53040*, *AT4G36600*, and *AT5G44310* are the ones that are both members of the ABI3 regulon and the LEA gene family. The remaining six (*AT1G27990*, *CBSX4*, *RTNLB13*, *AT2G25890*, *AT3G54940*, and *AT4G25580*) are members of the ABI3 regulon that are not LEA genes: *AT1G27990* encodes an unknown protein; *CBSX4* encodes a cystathionine-β-synthase (CBS) domain-containing protein involved in reproduction and is highly expressed in dry seeds (Fang et al. 2011); *RTNLB13* codes for a plant reticulon localized in the tubular endoplasmic reticulum and is predicted to be expressed in seeds (Sparkes et al. 2010); *AT2G25890* codes for a sugar-regulated oleosin (Huang et al. 2010); *AT3G54940* encodes a papain-like proteinase up-regulated in senescing siliques (Trobacher et al. 2006) and *AT4G25580* encodes a protein related with response to stress and is highly similar to a CAP160 protein linked with cold acclimation (Kaye et al. 1998).

**Fig. 8:**
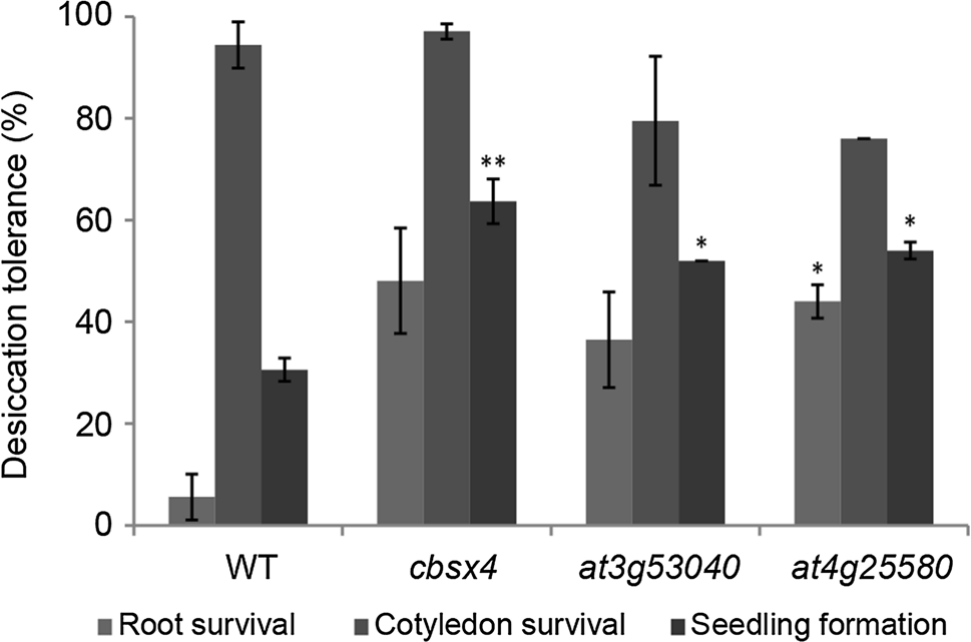
Re-establishment of desiccation tolerance in germinated seeds of wild type (WT) and T-DNA insertion lines scored as survival of primary root (root survival) and cotyledons and seedling formation. *Bars* represent standard error of three replicates. *Asterisks* indicate significant differences at *P* ≤ 0.05 for one and *P* ≤ 0.01 for two asterisks

For the phenotypic characterization in relation to re-establishment of DT, germinated seeds in the stage of appearance of first root hairs were used instead of germinated seeds in the stage of radicle protrusion. At the stage of radicle protrusion, germinated seeds of all the lines, including the wild-type, were able to re-establish DT in more than 90 % of germinated seeds (data not shown). Therefore, no differences were detected between them. On the other hand, at the stage of appearance of first root hairs, germinated seeds were not able to fully re-establish DT, allowing a clearer separation between wild-type seeds and seeds from T-DNA insertion lines. During evaluation of the survival, frequently, when the primary root fails to survive drying, the hypocotyl remains able to generate lateral roots, allowing normal seedling formation. Therefore, the combined evaluation of root survival, cotyledon survival and seedling formation provides a complete representation of the phenotypic influence of the T-DNA insertion on the re-establishment of DT. Three lines had a significant increase in the ability to re-establish DT compared to the wild-type: *cbsx4*, *at3g53040* and *at4g25580* (Fig. 8; Table 3). Three different lines had a decrease in seed vigor, with *at1g27990* being affected in high temperature seed vigor, and *at3g54940* and *at5g44310* showing severely reduced germination under salt stress (Table 3). The genes disrupted in these six lines had a fast increase in transcript level (expression level increase of more than fourfold) already after 2 h of incubation in ABA and remained highly expressed but without significant changes until the last time-point. None of the lines showed phenotypes for seed dormancy and longevity (Suppl. Table S3).

**Table 3:** Genes chosen for phenotypic characterization with T-DNA insertion lines and their phenotyping results

AGI	Annotation	Seed vigor		DT re-induction
		High temperature	Salt	
AT1G27990	Unknown protein	↓↓		
AT1G80090	CBS DOMAIN CONTAINING PROTEIN 4 (CBSX4)			↑↑
AT2G23640	RETICULAN LIKE PROTEIN B13 (RTNLB13)			
AT2G25890	Oleosin family protein			
AT2G36640	EMBRYONIC CELL PROTEIN 63 (ECP63)			
AT2G42560	LEA domain-containing protein			
AT3G15670	LEA family protein			
AT3G53040	LEA protein, putative			↑
AT3G54940	Papain family cysteine protease		↓	
AT4G25580	CAP160 protein			↑
AT4G36600	LEA protein			
AT5G44310	LEA family protein		↓	

Arrows indicate significant increase (↑) or decrease (↓) as compared to the wild type. Single arrows indicate *P* ≤ 0.05 and double arrows indicate *P* ≤ 0.01

## Discussion

Studies focusing on re-establishment of DT often use PEG alone or in combination with low temperature or ABA (Buitink et al. 2003; Vieira et al. 2010; Maia et al. 2011, 2014). In each case, seeds are exposed to a mild osmotic stress that often triggers a series of ABA-related responses (Huang et al. 2008; Matsui et al. 2008; Dalal et al. 2009). To evaluate the responses to ABA alone, without additional stressors, we studied germinated *A. thaliana* seeds at the stage of radicle protrusion during incubation in ABA. In this stage, DT can be re-established in all the seeds incubated in ABA already after 24 h of incubation (Fig. 1), while approximately 85 % of seeds incubated in PEG for 24 h had re-established DT (Maia et al. 2011). DT re-established in all the seeds by incubation in PEG is only obtained after 72 h of incubation (Maia et al. 2011).

In general, osmotic stresses trigger a series of physiological responses in ABA-dependent and ABA-independent manners with the existence of a cross-talk between them (Buitink et al. 2003; Yamaguchi-Shinozaki and Shinozaki 2005). A comparison between over-represented GO categories in DEGs with accumulating transcripts in response to incubation in ABA and in PEG reinforced the idea that these treatments lead to the re-establishment of DT through partially overlapping mechanisms.

A closer look at the main processes that occur during incubation in ABA revealed enrichment of different GO categories of biological processes (Fig. 4), attesting the growth arrest and the partial return to a quiescent stage similar to the dry seed (Buitink et al. 2006; Maia et al. 2011).

Lipid storage was the most over-represented GO category within the genes with accumulating transcript levels and the genes inside this category are oleosins and oleosin family proteins. Oleosins are typical for the later stages of seed development and, in desiccation tolerant seeds, they prevent lipid bodies from coalescing on dehydration and prevent the disruption of cellular structures during rehydration (Leprince et al. 1998; Pammenter and Berjak 1999).

Despite the fact that all the germinated seeds incubated in ABA had re-established DT after 24 h, the incubation was maintained for a total of 72 h, which improves longevity compared with incubation for 24 h (Fig. 2). Also during development, acquisition of DT precedes acquisition of longevity, likely to ensure timely response of stress-related genes (such as LEAs, HSPs and oxidative stress-related genes) that also improve seed storability (Verdier et al. 2013).

Transcriptomic studies generate large datasets. Visualization and analysis of this kind of data as networks is an important approach to explore a wide variety of biological relations (Freeman et al. 2007; Dekkers et al. 2013). To construct a network, the similarity between individual expression profiles may be determined and used as edges that connect nodes, or genes (Freeman et al. 2007). Once the network is given, an analysis of its structure can indicate key genes, functional modules or relations between the network structure and additional information (Villa-Vialaneix et al. 2013). Several centrality measures or topological indexes have been used to analyze networks. The degree centrality or just degree is thought to be simplest (Scardoni et al. 2009; Piraveenan et al. 2013; Bass et al. 2013). It refers to the number of links a given node has with other nodes and allows an immediate evaluation of the relevance of the node to the network (Scardoni et al. 2009; Piraveenan et al. 2013). Nodes with high degrees are called hubs and are thought to play important roles in organizing the behavior of the network (Dong and Horvath 2007; Bass et al. 2013; Villa-Vialaneix et al. 2013).

The ER region of the network contains genes related mainly to wax biosynthetic processes, lipid storage, seed development and response to abscisic acid stimulus. Most of the genes with accumulating transcript levels in relation with dormancy, DT acquisition, re-establishment of DT with PEG, and drought also locate in this region (Fig. 6). Also, all the hubs and most of the members of the ABI3 regulon, LEA proteins and TFs found in the network are in this region (Fig. 7). ABA is a key component of responses to abiotic stresses and regulation of seed dormancy and germination. The responses to abiotic stresses elicited by ABA include minimization of water transpiration (for example, by the accumulation of cuticular wax), and synthesis of protectants (such as LEA proteins) and antioxidants (Dalal et al. 2009; Seo et al. 2011). During seed development and germination, several genes under the control of ABA are related to the acquisition and loss of dormancy and DT (Toorop et al. 2005; Buitink et al. 2006; Maia et al. 2011; Terrasson et al. 2013). Moreover, ABA-responsive gene expression is directly regulated by TFs (Fujita et al. 2011). The TFs in the ER region of the network, such as ABI5, AP2/ERF TFs and NAC TFs, are known to be related with response and tolerance to abiotic stress possibly by a rapid amplification and broadening of signal responses (Fujita et al. 2011). Taken together, these observations reinforce the relation between early stress response and seed development and the view that early responsive genes may provide initial protection and amplification of signals (Buitink et al. 2006).

The LR region contains genes related mainly with response to abiotic stimulus (especially light stimulus), and aromatic amino acid family metabolic processes. The GO category aromatic amino acid family metabolic processes refer to chemical reactions and pathways involving amino acids with an aromatic ring, such as phenylalanine, tyrosine, and tryptophan, which could be involved in protein stability (Khuri et al. 2001; Carbon et al. 2009). TFs located in this region are related to abiotic stress tolerance, growth regulation and light signaling pathways and might influence the plant’s ability to adapt to daily changes in water status in a coordinated action with the circadian clock (Alabadí et al. 2001; Fujita et al. 2011). These results are in agreement with the hypothesis that genes which are responding later may be involved in adaptation to stress conditions (Buitink et al. 2006).

We considered the 100 nodes with the highest degrees as hubs. They are all concentrated in the ER region and most of them follow the expression pattern corresponding to the 5th clustered pattern of the SOM analysis (fast increase in expression in the first 2 h of incubation in ABA followed by certain stabilization in the following hours).

Three T-DNA insertion lines showed a higher ability to re-establish DT compared to wild-type, suggesting the existence of de-repression mechanisms and redundancy between highly similar genes. These lines are *cbsx4*, *at3g53040* and *at4g25580*. CBSX4 is one of the two *A. thaliana* homologous genes (the other is AT1G15330) that encode CBS domain-containing proteins that belongs to the PV42 class of gamma subunits of SnRK1 (Fang et al. 2011). CBS domain-containing proteins have been found to act in a variety of biological processes, such as metabolic enzymes, transcriptional regulators, ion channels, and transporters (Rosnoblet et al. 2007; Fang et al. 2011). *A. thaliana* mutants in *HISTONE ACETYLTRANSFERASE1* (*HAC1*) had reduced expression of both *AT1G15330* and *CBSX4*, suggesting a role of these genes in sugar sensing and fertility (Heisel et al. 2013). Phenotyping a T-DNA insertion line for *AT1G15330* did not show any phenotypes (data not shown). The observed phenotype of higher ability to re-establish DT compared to wild-type seeds could be due to compensatory effects of one homologous gene over the other. Interestingly, *cbsx4* had an increased expression of the mutated gene compared to the wild-type in a RT-PCR experiment. We speculate that the amplification of this gene generates a product with a non-functional sub-unit, as the presence and location of the insertion in the last exon of the gene was confirmed.

*AT3G53040* codes for a cytosolic LEA domain-containing protein that shares typical features of the LEAM protein class A α-helix motifs, being classified as LEA_4 family (Candat et al. 2014). Proteins of this family are likely to interact and protect various cellular membranes during dehydration (Candat et al. 2014). In *A. thaliana* seedlings, the expression of *AT3G53040* was shown to respond to ABA, but without inducible expression to stress conditions (Huang and Wu 2006; Bies-Ethève et al. 2008). *AT3G53040* and *AT2G36640* are very similar to each other in sequence and expression pattern and are considered to form a pair derived from a whole genome duplication (Hundertmark and Hincha 2008; Bies-Ethève et al. 2008). It is expected that duplicated genes have redundant functions and the higher ability to re-establish DT observed in *at3g53040* compared to wild-type could be caused by this redundancy and compensatory effects. It is likely that the phenotyping of a T-DNA insertion line of *AT2G36640* did not result in differences compared to the wild-type (data not shown) due to this redundancy.

*AT4G25580* codes for a stress-responsive protein-related with weak similarity (less than 20 % identity) with LEA group 2 (Bies-Ethève et al. 2008) and high similarity to a CAP160 protein (Mönke et al. 2012) and to a CDeT11-24 from *Craterostigma plantagineum* (Röhrig et al. 2006). In spinach, CAP160 proteins are predominantly cytosolic, induced by drought stress and related to stabilization of membranes, ribosomes and cytoskeletal elements (Kaye et al. 1998). CDeT11-24 is thought to contribute to the plant’s DT possibly by interacting with other proteins such as dehydrins (Röhrig et al. 2006). However, *at4g25580* seeds had an increased ability to re-establish DT compared to wild-type seeds, suggesting the operation of derepression mechanisms.

None of the T-DNA lines analyzed showed phenotypes for seed dormancy or longevity. Only one of the analyzed genes (*AT3G53040*) was reported to be differentially expressed comparing dormant and after-ripened states (Cadman et al. 2006; Bassel et al. 2011). Considering the increase in longevity between 24 and 72 h of incubation in ABA and the location in the network (ER region) of the genes chosen for phenotypic characterization, it is conceivable that the disruption of single genes would not influence longevity. Besides, as only three of the T-DNA lines had the vigor affected, we believe that seed development was not significantly affected by the disruption of the single genes.

Taken together, these results confirm that the analysis of co-expression network structures can bring insights to biological processes. We showed that the re-establishment of DT in germinated *A. thaliana* seeds can be divided into two phases. In the first phase, a series of stress-responsive genes that are also related to seed development as well as other biological processes (such as dormancy, acquisition of DT, drought and the circadian clock) is induced, promoting amplification of signals, growth arrest and protection mechanisms (such as LEA proteins). In the second phase, photosynthesis and primary metabolism are strongly inhibited and another set of stress-responsive genes promotes adaptation to stress conditions that also contribute to seed survival in the dry state, improving longevity. Moreover, we suggest that redundancy and compensatory mechanisms may be operating when genes important for DT are disrupted.

### *Author contribution statement*

MCDC designed research, conducted experiments, analyzed data and wrote the manuscript. KR analyzed data and wrote the manuscript. HN designed scripts for microarray analysis. FY conducted experiments. WL, JB and HWMH conceived and designed research, analyzed data and wrote the manuscript. All authors read and approved the manuscript.

## Electronic supplementary material

Below is the link to the electronic supplementary material.

Supplemental Fig. S1 Schematic illustration and relative abundance of transcripts of mutated genes in *cbsx4*, *at3g53040* and *at4g25580*. **a** Mapped T-DNA insertion sites (triangles) are indicated on the top of the genomic structure. Black boxes on the black solid line indicate the exons, gray boxes indicate 3′and 5′ untranslated regions and arrows indicate location of qPCR primers (fwd: forward; rev: reverse). **b** Normalized relative expression levels of transcripts of mutated genes in *cbsx4*, *at3g53040* and *at4g25580* compared to wild-type (WT) calculated with the qBase software (Hellemans et al. 2007). Asterisks indicate significant differences at *P* ≤ 0.05 for one, *P* ≤ 0.01 for two asterisks (TIFF 220 kb)

Supplemental Fig. S2 Temporal expression profiles of 20 genes measured by qPCR and microarray after incubation of germinated seeds in the stage of radicle protrusion for 0 h, 2 h and 24 h. Blue lines indicate expression levels measured by qPCR and red lines indicate expression levels measured by microarray (TIFF 551 kb)

Supplementary material 3 (XLSX 261 kb)

## Abbreviations

ABA: Abscisic acid
DT: Desiccation tolerance
DEG: Differentially expressed gene
ER: Early response
GO: Gene ontology
HSP: Heat shock protein
LR: Late response
LEA: Late embryogenesis abundant
ORA: Over-representation analysis
PEG: Polyethylene glycol
SOM: Self-organizing map
TF: Transcription factors
