# Supplementary figures and images for: A gene co-expression network predicts functional genes controlling the re-establishment of desiccation tolerance in germinated Arabidopsis thaliana seeds
Source: Planta. 2015 Mar 26;242(2):435–49. doi: 10.1007/s00425-015-2283-7 (PMC4498281; doi:10.1007/s00425-015-2283-7)

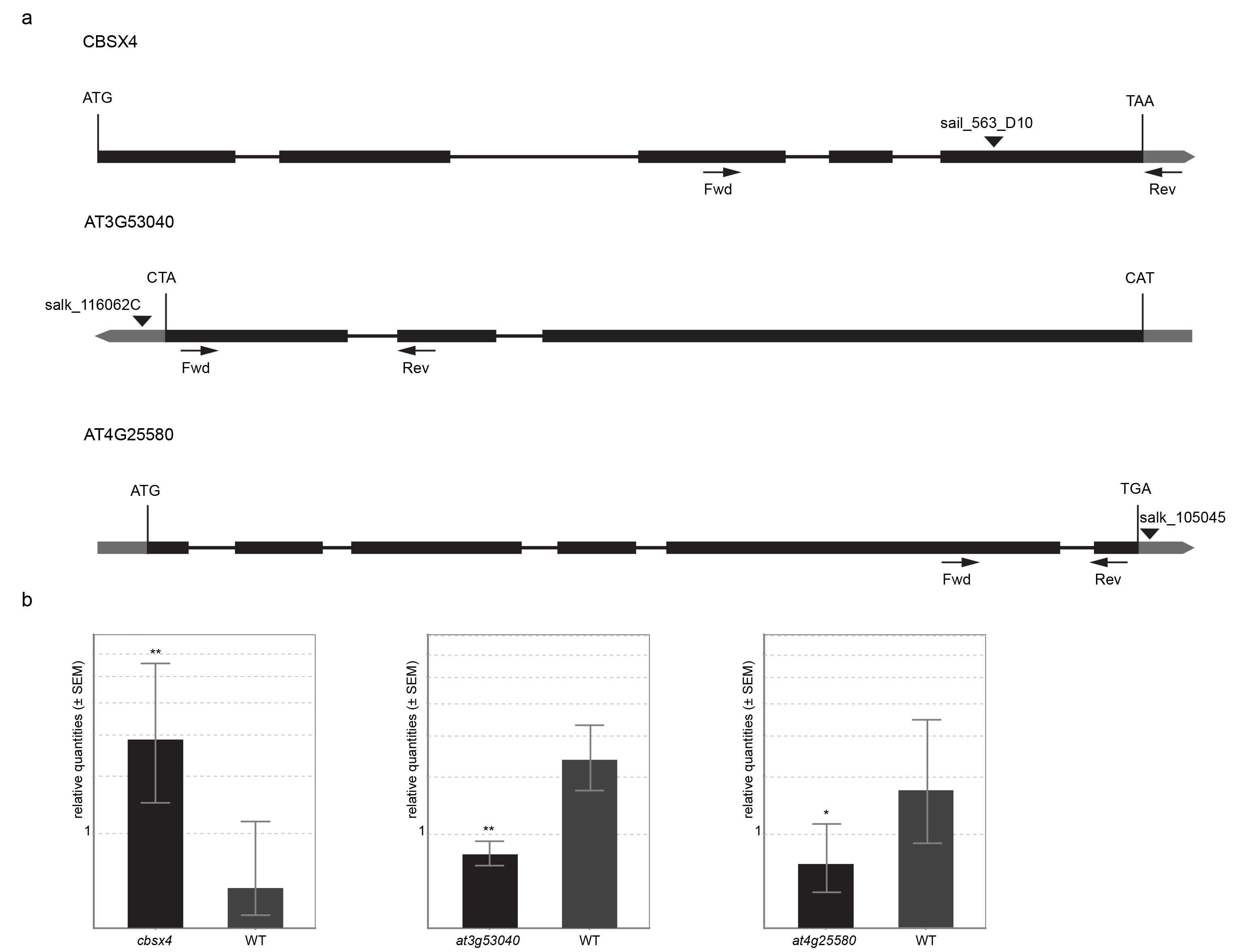

Supplement: Supplementary file 1 — Supplemental Fig. S1 Schematic illustration and relative abundance of transcripts of mutated genes in cbsx4, at3g53040 and at4g25580. a Mapped T-DNA insertion sites (triangles) are indicated on the top of the genomic structure. Black boxes on the black solid line indicate the exons, gray boxes indicate 3′and 5′ untranslated regions and arrows indicate location of qPCR primers (fwd: forward; rev: reverse). b Normalized relative expression levels of transcripts of mutated genes in cbsx4, at3g53040 and at4g25580 compared to wild-type (WT) calculated with the qBase software (Hellemans et al. 2007). Asterisks indicate significant differences at P ≤ 0.05 for one, P ≤ 0.01 for two asterisks (TIFF 220 kb) [file 425_2015_2283_MOESM1_ESM.tif]

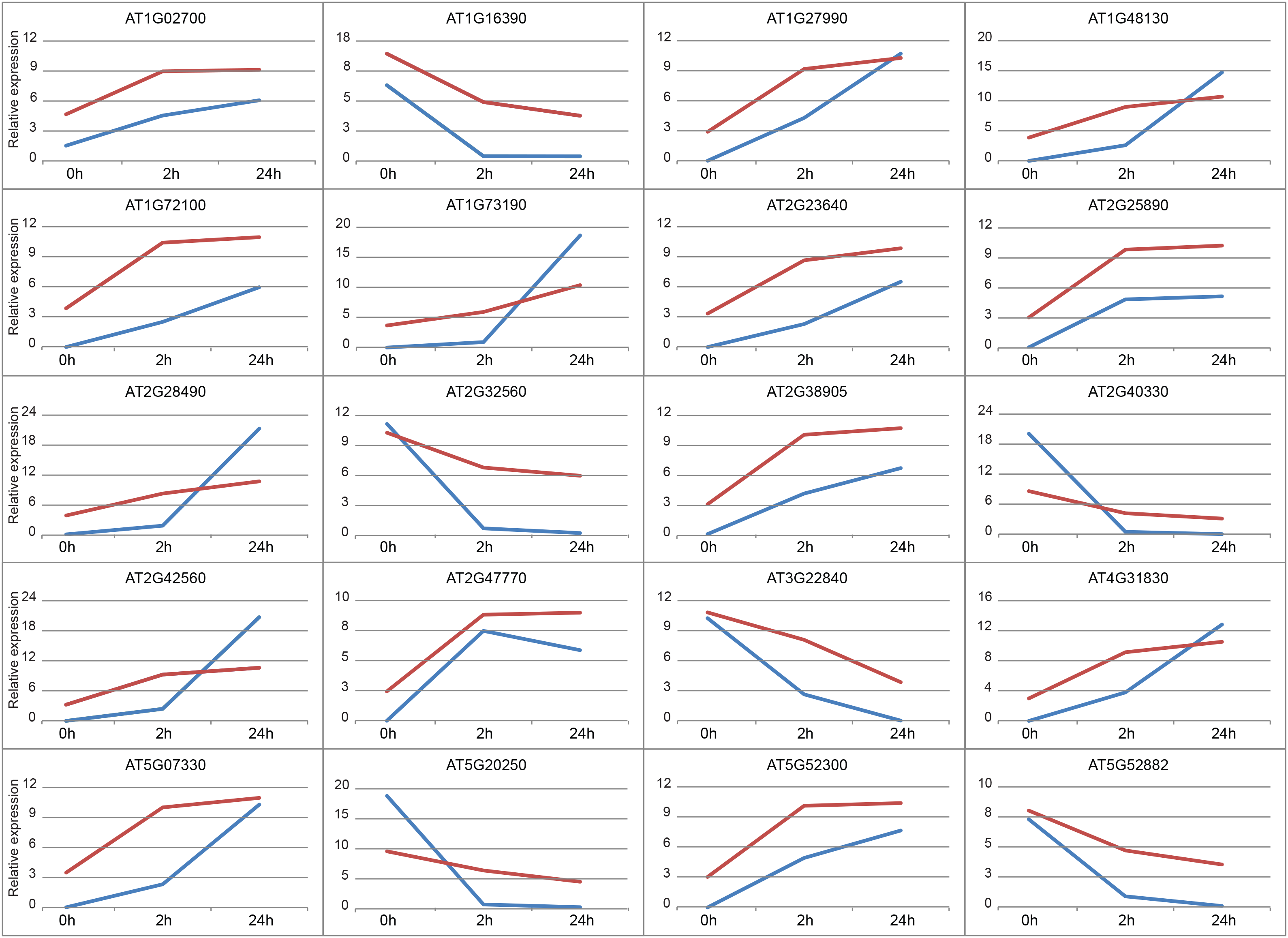

Supplement: Supplementary file 2 — Supplemental Fig. S2 Temporal expression profiles of 20 genes measured by qPCR and microarray after incubation of germinated seeds in the stage of radicle protrusion for 0 h, 2 h and 24 h. Blue lines indicate expression levels measured by qPCR and red lines indicate expression levels measured by microarray (TIFF 551 kb) [file 425_2015_2283_MOESM2_ESM.tif]
